# Pelvic Fixation Technique Using the Ilio-Sacral Screw for 173 Neuromuscular Scoliosis Patients

**DOI:** 10.3390/children11020199

**Published:** 2024-02-04

**Authors:** Mathilde Gaumé, Elie Saghbiny, Lou Richard, Clélia Thouement, Raphaël Vialle, Lotfi Miladi

**Affiliations:** 1 University Institute for Spine Surgery, Armand Trousseau Hospital, Sorbonne University, 26 Avenue du Dr Netter, 75012 Paris, France; mathilde.gaume@aphp.fr (M.G.); elie.saghbini@aphp.fr (E.S.); lou.richard@aphp.fr (L.R.); clelia.thouement@aphp.fr (C.T.); 2 Pediatric Orthopedic Surgery Department, Necker Hospital, APHP, University of Paris-Cité, 75015 Paris, France; l.miladi@aphp.fr

**Keywords:** neuromuscular scoliosis, pelvic fixation, pelvic obliquity, minimally invasive fusionless surgery, ilio-sacral screw, posterior spinal fusion

## Abstract

Pelvic fixation remains one of the main challenging issues in non-ambulatory neuromuscular scoliosis (NMS) patients, between clinical effectiveness and a high complication rate. The objective of this multicenter and retrospective study was to evaluate the outcomes of a technique that was applied to treat 173 NMS patients. The technique is not well-known but promising; it uses the ilio-sacral screw, combined with either the posterior spinal fusion or fusionless bipolar technique, with a minimum follow-up of two years. The mean operative age of the patients was 13 ± 7 years. The mean preoperative main coronal curve was 64° and improved by a mean of −39° postoperatively. The mean preoperative pelvic obliquity was 23°, which improved by a mean of −14° postoperatively. No decrease in the frontal or sagittal correction was observed during the last follow-up. The sitting posture improved in all cases. Twenty-nine patients (17%) had a postoperative infection: twenty-six were treated with local debridement and antibiotics, and three required hardware removal. Fourteen mechanical complications (8%) occurred: screw malposition (*n* = 6), skin prominence (*n* = 1), and connector failure (*n* = 1). This type of surgery is associated with a high risk for infection. Comorbidities, rather than the surgery itself, were the main risk factors that led to complications. The ilio-sacral screw was reliable and effective in correcting pelvic obliquity in NMS patients. The introduction of intraoperative navigation should minimize the risk of screw misplacement and facilitate revision or primary fixation.

## 1. Introduction

Scoliosis is a deformity in all three planes of the spine that can occur at any stage of life. It may be idiopathic, secondary, or degenerative. Neuromuscular scoliosis can occur in patients with any type of pre-existing neuromuscular diagnosis; it is characterized by rapid worsening during growth and may continue to progress after skeletal maturity [1]. Neuromuscular scoliosis can be caused by a disorder of the brain, central or peripheral motor neurons, or muscular system. Intellectual disability and digestive, cardiac, and respiratory problems can also be associated with neuromuscular diseases. Fixed pelvic obliquity, defined as an angulation of the pelvis relative to the horizontal axis in the frontal plane, is frequently associated with spinal deformity and can lead to difficulties in maintaining a good sitting position, the onset of pain while sitting, and skin breakdown [2,3]. Conservative treatment with bracing and serial casting is poorly tolerated and has been proven to be limited [4,5]. As a result, surgery becomes necessary very quickly, with the aim to obtain a stable, compensated spine, with a balanced trunk control level [6,7]. The long posterior spinal fusion technique, using pedicular screws, or minimally invasive fusionless surgery [8], are currently the two main surgical options. Posterior vertebral arthrodesis is the gold standard of treatment for neuromuscular scoliosis, but this surgery requires skeletal maturity and is unsatisfactory before puberty [9]. In younger children, growth-sparing surgery has been developed to stabilize the spine while preserving the growth of the spine and of the thoracic cage, and postponing arthrodesis. Minimally invasive fusionless surgery is an original growth-sparing technique based on a bipolar telescopic construction whose main strength is not only to preserve spinal and thoracic growth but also to avoid arthrodesis at skeletal maturity [10].

In non-ambulatory patients, pelvic fixation is performed to achieve coronal and sagittal alignment in cases of pelvic obliquity greater than 15° or in cases of low lumbar curvature [11]. However, pelvic fixation in neuromuscular patients increases the technical difficulties and the risk of both pseudarthrosis and skin breakdown, because of their very fragile general condition and poor bone quality. These patients require multidisciplinary care in a specialized center to ensure that the procedure is planned after respiratory, nutritional, and orthopedic preparation. The difficulty in finding the best option is reflected in the wide variety of pelvic fixations described in the literature [12,13,14,15]. Modern pelvic fixation techniques include sacral, iliac, sacral alar iliac, and ilio-sacral screws. All these types of fixation techniques have their advantages and disadvantages, but what they have in common is that they all have many mechanical complications.

**Figure 1:**
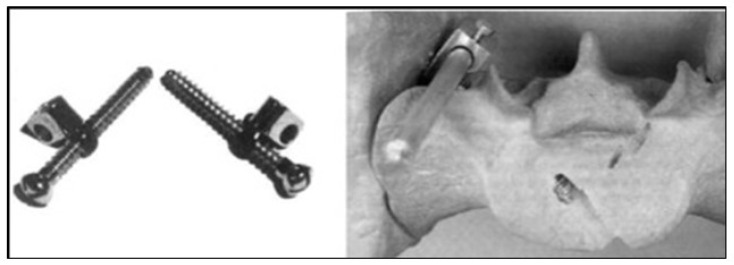
The ilio-sacral screw with the connector.

The ilio-sacral screw was introduced as one of Cotrel–Dubousset sacral instruments, which also include alar staples and sacral screws [16], owing to the design of a double connector by Jacques Beurier. Pelvic fixation improves the corrective lever arm and bony purchase by extending to the S1 pedicle through the two cortices of the posterior ilium [17] (Figure 1). This technique has been proven to be effective in retrospective and prospective neuromuscular scoliosis studies, with a high rate of pelvic obliquity correction (from 39.1 to 84%) and reduced rates of lumbosacral pseudarthrosis (0–0.65%) [18,19].

The objective of the following study was to validate the reliability of the ilio-sacral screw pelvic fixation technique using either posterior spinal fusion or minimally invasive fusionless surgery in a large series of neuromuscular scoliosis patients.

## 2. Materials and Methods

### 2.1. Population

A retrospective review of all consecutive non-ambulatory patients with neuromuscular scoliosis who underwent either posterior spinal fusion or minimally invasive fusionless surgery with a minimum follow-up of 2 years was conducted, including 173 patients in two academic children’s hospitals (Paris, France), which are also referral centers for neuromuscular disorders. Patients who were diagnosed with neuromuscular scoliosis associated with pelvic obliquity >15° and requiring major surgery extending to the pelvis with fixation by the ilio-sacral screw were included. Patients were excluded if the scoliosis was not of neuromuscular origin or if prior spinal surgery or pelvic fixation was performed.

### 2.2. Operative Techniques

**Figure 2:**
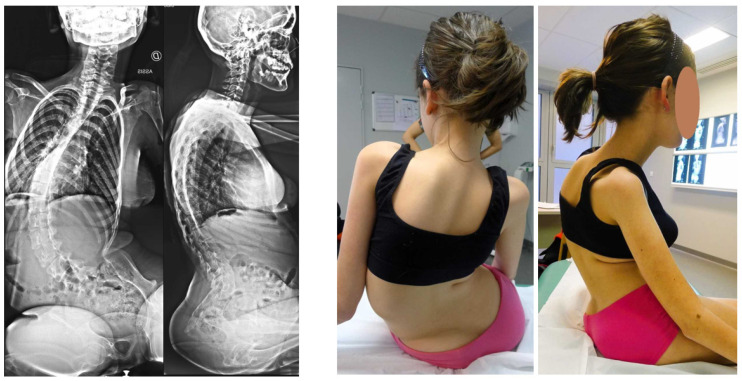
Preoperative X-rays and clinical pictures of a SMA2 patient.

**Figure 3:**
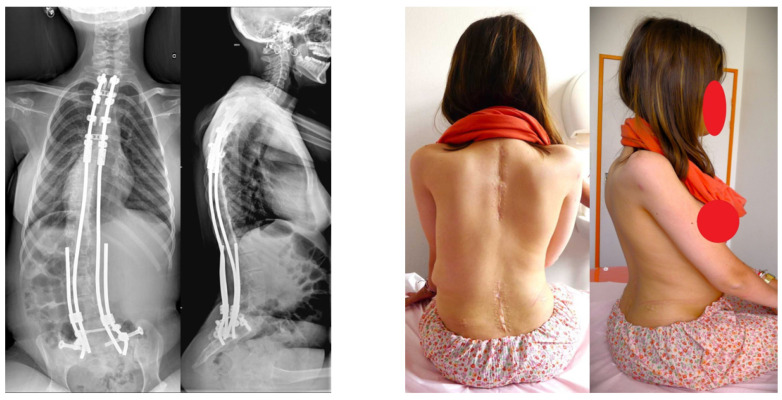
Postoperative X-rays and clinical pictures of the same patient, after minimally invasive fusionless surgery.

**Figure 4:**
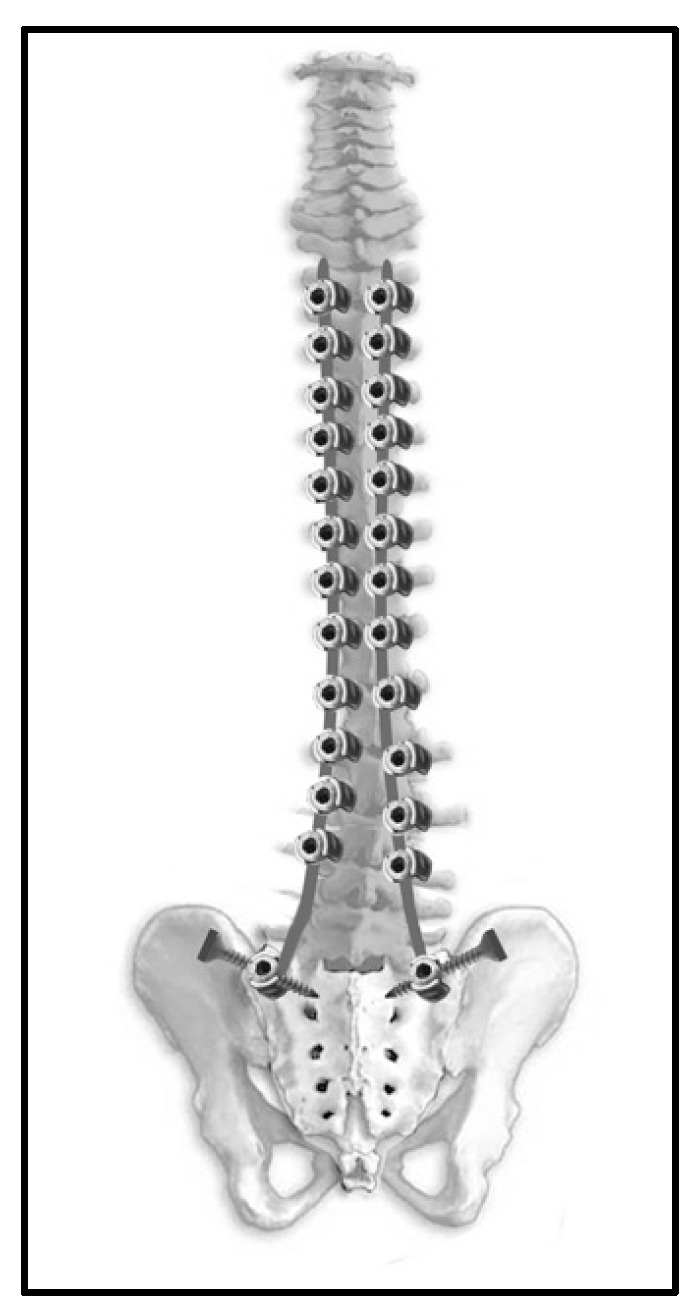
Posterior spinal fusion with ilio-sacral screws.

Patients whose triradiate cartilage was still open underwent minimally invasive fusionless surgery (minimally invasive fusionless surgery group, Figure 2 and Figure 3), and those in whom it was fused were managed using posterior spinal fusion (posterior spinal fusion group, Figure 4).

Spinal surgery was performed by the same senior surgeon for all minimally invasive fusionless surgeries (Lotfi Miladi) and all posterior spinal fusion patients (Raphael Vialle). The patient was placed in the prone position in all cases.

**Figure 5:**
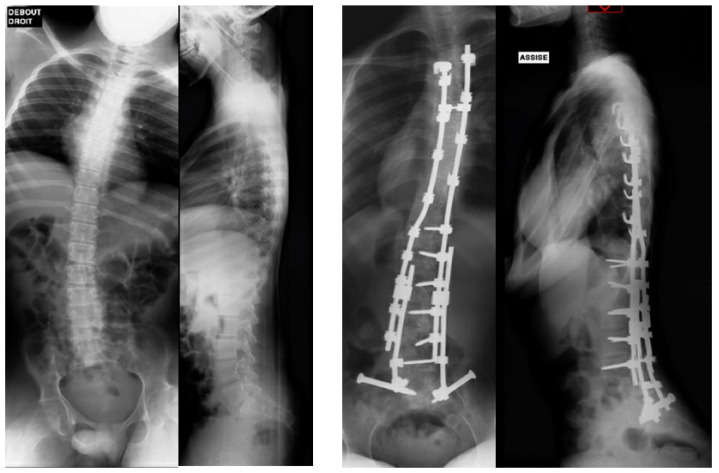
Pre- and postoperative X-rays after posterior spinal fusion.

Posterior spinal fusion was performed using a posterior approach with spinal correction and fusion using pedicle screws, pedicle hooks, and transverse hooks at the first proximal thoracic level. The surgical technique used for the placement of the ilio-sacral screw in posterior spinal fusion procedures has been described by Zahi et al. [18]. The ilio-sacral screws were connected to the two short rods. Frontal pelvic obliquity was corrected using distraction and contraction maneuvers between the long and short rods (Figure 5).

The minimally invasive fusionless surgery was performed with two small midline skin incisions: the first was centered on the upper thoracic spine, and the second was centered on the lumbosacral junction. Proximal bilateral fixation was achieved at the first thoracic vertebrae using a double claw of the supralaminar and pedicle hooks attached to two adjacent vertebrae and separated by a free vertebra. A rod system composed of two long pre-curved rods was inserted intramuscularly from the proximal incision to the distal incision and attached to the proximal hooks. These rods were retained medially and attached using a side-to-side closed connector to an overlapping shorter lateral rod attached to the distal multiaxial connector of the ilio-sacral screw at S1. The amount of overlap between the two rods corresponded to the required lengthening potential of the construct. To create the final stable frame construct, cross-links were used proximally and distally.

The surgical technique used for placement of the ilio-sacral screw in minimally invasive fusionless surgery has been described by Miladi et al. [8]. The Wiltse approach allows for access to the lumbosacral joint after a short midline lumbo-pelvic incision. The posterior sacral cortex is exposed medially from the L5–S1 joint to the sacral ala, laterally and distally from the first posterior sacral foramina. A trough was made lateral to the L5–S1 joint and above the first posterior sacral foramen. The ilio-sacral connector was fixed using a connector holder and inserted into the trough. A guide was attached to the connector holder to facilitate screw insertion. Once the guide was removed, the cannulated screw was inserted percutaneously through the ilio-sacral connector from the posterior part of the iliac wing towards the sacrum to reach the body of S1. The screw was then locked within the ilio-sacral connectors.

The screw length can vary. The sizes were selected based on the anatomical parameters of the preoperative films. Fully threaded ilio-sacral screws were implanted freehand.

Postoperative management was performed for a few days in the intensive care unit, followed by management in a traditional orthopedic ward. Rehabilitation was performed at home or at a rehabilitation center.

### 2.3. Outcomes Assessment

Age and etiology of the neuromuscular disorder were recorded as demographic data.

Radiographs were obtained in the sitting position before surgery, after surgery, and at the last follow-up [20]. Measurements were obtained using PACS-Carestream software 12.0 (Rochester, NY, USA: Carestream Health). The Cobb angle of the main curve and pelvic obliquity were measured. The Cobb angle was measured by drawing lines parallel to the upper border of the upper vertebral body and the lower border of the lowest vertebra of the structural curve, and then by drawing perpendicular lines from these lines to cross each other. Pelvic obliquity was defined as “the angle subtended by a line drawn between the most proximal points of the iliac crest and a line drawn parallel to the lower end of the roentgenogram” [21].

Complications including surgical site infections and the mechanical failure of the ilio-sacral screw were recorded.

Patients and/or caregiver-reported outcome questionnaires were also used to assess sitting posture (same, better, or worse) and comfort improvement (same, better, or worse). Pain was rated from 0 to 10, with 0 indicating the absence of pain and 10 indicating the maximum pain.

### 2.4. Statistics

Data were processed using SPSS V23 software (IBM Corporation, Armonk, NY, USA). Comparisons between the two groups were performed using Fisher’s exact test or the chi-squared test. A comparison of deformity correction was performed using a non-parametric analysis of paired samples. Statistical significance was set at *p* < 0.05.

## 3. Results

### 3.1. Demographic Data

A total of 173 patients were included, and none were lost to follow-up. Posterior spinal fusion was performed in 62 patients and minimally invasive fusionless surgery was performed in 111 patients. All patients were non-ambulatory. The mean operative age was 13.7 ± 7 years (12 in the minimally invasive fusionless surgery group and 15 in the posterior spinal fusion group). The etiologies included cerebral palsy (*n* = 113), spinal muscular atrophy (*n* = 19), muscular dystrophy (*n* = 14), flaccid paraplegia (*n* = 11), myelomeningocele (*n* = 9), and Rett syndrome (*n* = 7). Significantly more patients had cerebral palsy and myelomeningocele in the minimally invasive fusionless surgery group (*p* < 0.05).

### 3.2. Radiological Data

The differences between the main curve deformity and pelvic obliquity for both groups preoperatively and at the latest follow-up are reported in Table 1. The preoperative major curve was higher in the minimally invasive fusionless surgery group (77.0 vs. 50.5°). Preoperative pelvic obliquity was not significantly greater in the two groups. Before surgical correction, the mean Cobb angle of the main coronal curve was 63.8°, which improved by a mean of −39.5° (−60%) postoperatively. Mean preoperative pelvic obliquity was 23.0°, which improved by a mean of −14° postoperatively (61%). Pelvic obliquity was better corrected in the minimally invasive fusionless surgery group. No loss of frontal or sagittal correction was observed at the last follow-up.

### 3.3. Complications

Fourteen mechanical complications (8%) occurred: the early mobilization of one ilio-sacral screw made it necessary to change the screw for a longer one percutaneously (*n* = 6); screw malposition with S1 root irritation (*n* = 6); screw skin prominence (*n* = 1); and connector failure (*n* = 1). There were no significant differences in mechanical complications between the minimally invasive fusionless surgery and posterior spinal fusion groups.

Twenty-nine patients (17%) had an early postoperative infection, with favorable outcomes in the 26 patients who were treated with local wound debridement and antibiotics. In three cases, a persistent chronic Staphylococcus aureus infection required hardware removal. The outcome was favorable in all patients, with satisfactory healing and no recurrence of infection following hardware removal. On average, the patients waited one year before being re-instrumented.

### 3.4. Quality of Life

According to the patients and/or caregivers, sitting posture and comfort were qualified as “better” in all cases after surgery, with a clear improvement in transfers in daily life, particularly from bed to chair.

The only patients who described postoperative pain were due to S1 nerve root irritation, associated with ilio-sacral screw malposition. The pain was relieved once the screw path was corrected.

## 4. Discussion

This study focused on the outcomes of patients with neuromuscular scoliosis who underwent either posterior spinal fusion or minimally invasive fusionless surgery with pelvic extension using ilio-sacral screws. This is the largest series described in the literature that compares ilio-sacral screws in the same cohort. The results demonstrate that fixation using ilio-sacral screw is effective in pediatric patients with neuromuscular scoliosis, with a 60% correction of the Cobb angle and a 61% correction of pelvic obliquity, respectively. Pelvic obliquity correction was better in the minimally invasive fusionless surgery group than in the posterior spinal fusion group because of repetitive surgeries for lengthening procedures in cases of major residual pelvic deformity [8]. The lengthening procedure was performed with a previous distal incision for access to the side-to-side connectors and the possibility of the asymmetrical lengthening of the rods.

The present results are consistent with the current literature, with a 77% correction of pelvic obliquity and a 52% correction of the Cobb angle in a consecutive series of 167 neuromuscular scoliosis patients who exclusively underwent minimally invasive fusionless surgery with ilio-sacral screw pelvic fixation. Sixteen mechanical complications in nine patients happened: screw prominence (*n* = 1), connector failure (*n* = 4), and screw malposition (*n* = 11). Unplanned surgery was required in seven cases; two cases were managed during rod lengthening, and seven did not require treatment [22]. Miladi et al. [17] reported a correction of the Cobb angle of the main curve ranging from 53% to 70%, and the correction of pelvic obliquity ranging from 60% to 84%, in a series of 154 patients with neuromuscular scoliosis who underwent posterior spinal fusion with the ilio-sacral screw. Twenty-five complications were observed in seven patients, including four dislodgments of the ilio-sacral screw. The complications were caused by an infection in three patients and by a failure to check the tightness of the screw in one patient. Lumbosacral pseudarthrosis occurred in one patient, whereas none were reported in our series. In the literature, the pseudarthrosis rate in neuromuscular scoliosis patients ranges from 1.8 to 15% [23,24,25].

The literature on adult spinal deformity provides further insight into the effects of pelvic fixation with ilio-sacral screws in neuromuscular scoliosis patients. Wolff et al. [26] examined the outcomes in 15 adults with neuromuscular scoliosis who underwent minimally invasive fusionless surgery from the thoracic spine to the pelvis. Significant improvements in pain and balance were reported in all the patients. Only one connector failure was reported because of an inappropriate choice of the implant, which was too small (pediatric instead of adult shape).

The correction rate was also comparable to that of other pelvic fixation options, with a 63% mean correction of the Cobb angle of the main curve, and a 55% mean correction of pelvic obliquity with the sacral alar iliac fixation technique reported in the series by Jain et al. [27]. Sponseller et al. [28] compared the two-year postoperative radiographic parameters of 32 pediatric patients who underwent the procedure with the sacral alar iliac fixation technique and 27 patients who underwent the procedure with the sacroiliac technique. Among the patients who received the procedure with the sacral alar iliac fixation technique, the mean correction of pelvic obliquity was 70% and the mean Cobb angle correction was 67%. Among the patients who received the procedure using the sacroiliac technique, the values were 50% and 60%, respectively. Compared with other traditional techniques, sacral alar iliac screws provided a significantly better correction of pelvic obliquity, but no difference in the Cobb angle correction of the main curve or complications were observed.

We observed a rather high infection rate of 17%, which, however, seems to be within the range of previous reports in the literature (from 6 to 20%) [29,30]. Surgical site infections were more frequent in the minimally invasive fusionless surgery group than in the posterior spinal fusion group. The surgical approach may not influence the incidence of infection. For example, the correction of pelvic obliquity using the “T construct” for pelvic fixation requires an extensive dissection of the tissue at the caudal end of the spine to insert the horizontal portion of the “T”, with a similar infection rate of 18% in a series of 60 neuromuscular scoliosis patients [31]. Due to poor nutrition, poor wound healing, incontinence, and impaired communication, neuromuscular scoliosis procedures are known to be associated with frequent postoperative infections [32,33,34]. The incidence of infection in the minimally invasive fusionless surgery group may also be related to the possibility of iterative rod lengthening, with an increased infection risk after each procedure. The advent of one-way self-expanding rods, which are designed to avoid repeated surgery due to their free rod-sliding capabilities, should reduce the surgical site infection rate of minimally invasive fusionless surgeries in further studies. The infection rate decreased to 9% in a preliminary report of a prospective series of 21 patients who underwent procedures using one-way self-expanding rods with distal fixation using ilio-sacral screws after a minimum follow-up of three years. No complications related to the ilio-sacral screws have been reported [35].

In the present study, the mechanical complication rate was 8%, which is consistent with other types of pelvic fixation procedures in the literature. Procedures using the S2 alar iliac screw fixation technique have a 4 to 7% implant failure rate, and similar revision rates for implant failure [12,36,37].

The most common complication related to the use of ilio-sacral screws in the present series was S1 root irritation due to screw malposition (3.4%), which was also the most common reason for revision. However, these malpositions occurred before systematic preoperative control using 3D CT scanning. Notably, screw loosening was observed radiographically in a few X-rays with an osteolytic zone around the ilio-sacral screw <5 mm, but with no clinical or mechanical consequences in any patient. The risk of malposition is not just a problem for ilio-sacral screws, but also for other types of pelvic fixation, with various rates across studies. In adults, the breakthrough rate of procedures using the S2 alar iliac screw was 18% [38]. In Hassan et al.’s [39] pediatric cohort of 25 patients, screw breakthrough involving the lateral iliac-wing cortex occurred in eight (32%) patients. Eight percent of the patients had screw malposition, as confirmed by a postoperative CT scan. In contrast, Sponseller et al. [28], who has promoted the S2 alar iliac screw fixation technique in the pediatric population, reported no cases of malposition.

A recent computed tomography study demonstrated the ideal ilio-sacral screw trajectory to avoid malpositioning in children with non-neuromuscular scoliosis, which differs from that in adults. The mean optimal angles were 32.3° ± 3.6°, 33.8° ± 4.7°, 30.2° ± 5.0°, and 30.4° ± 4.7° in females < 10 years old, males < 10 years old, females > 10 years old, and males > 10 years old, respectively. The mean optimal angle differed between the two age groups (*p* = 0.004) but not between females and males (*p* = 0.55). The mean optimal screw length was 73.4 ± 9.9 mm. The transverse spinal canal anatomical parameters varied with age (*p* = 0.02) and sex in older children (*p* = 0.008), and the sagittal parameters varied with sex (*p* = 0.04) [40]. Such computed tomography studies should be of interest for patients with neuromuscular scoliosis.

Although the ilio-sacral screw technique is operator-dependent with a relatively longer learning curve than other pelvic fixation techniques, the emergence of new ancillaries should improve the accuracy of ilio-sacral screw positioning in the future. Moreover, innovative navigation methods and augmented reality surgery-like conditions could be interesting new teaching tools and surgical aids to enhance visualization and improve patient outcomes [41,42,43].

The ilio-sacral screw also has other strong advantages, particularly the low profile of the implant and its deep location, which permits a decrease in the risk of implant prominence and skin ulceration, which was reported in only one very skinny patient weighing <20 kg. The ilio-sacral screw can also be placed without exposing the iliac crest and without the potential devascularization of the overlying soft tissues, which may reduce complications due to implant prominence. The sacral alar iliac technique also has the key advantage of eliminating the need for subcutaneous muscle dissection over the iliac crest and decreasing the risk of implant prominence. In contrast, screw prominence reached over 11% with the iliac screw fixation technique [11].

The absence of pain in the sitting position after ilio-sacral screw surgery may be due to the screw path not crossing the sacroiliac joint and the tightening of the ilio-sacral screw perpendicular to the joint plane, allowing for a protective syndesmosis effect on the sacroiliac joint. Crossing the sacroiliac joint in childhood could lead to long-term pain, which has not yet been well evaluated, particularly with the S2 wing screw technique. Multiple pedicular screw fixation improves spinal rigidity in posterior spinal fusion constructs. Under these conditions, patients had an improved postoperative course with earlier mobilization and return to a comfortable sitting position. The improvement in spinal stabilization over time permitted a reduction in the need for a postoperative cast or brace in a minimally invasive fusionless surgery construct.

The absence of screw pullout could be due to the rod connection in the center of the screw and the perpendicular position of the screw to the iliac crest, crossing both cortices and ending transversely in the S1 body.

Our study had several limitations that should be addressed in future studies. First, it was retrospective and based on reported findings. There were no comparisons or randomized groups with other pelvic fixation techniques. It was also difficult to assess functional outcomes in our patients. This is particularly true for patients with cerebral palsy and severe intellectual disability [44]. Finally, the effect of posterior spinal fusion or minimally invasive fusionless surgery with the ilio-sacral screw pelvic fixation technique in children with ambulatory ability has not yet been evaluated. Drake et al. published the results of a large study of 118 patients with neuromuscular scoliosis, including 11 ambulatory patients with pelvic extension using either sacral alar iliac or iliac screws [45]. They found that all patients were able to walk with the same or better function after posterior spinal fusion with pelvic extension. In addition, in terms of hardware failure, no significant difference was found between the ambulatory and non-ambulatory groups.

Despite these limitations, this study is the first in the literature to evaluate the effect of ilio-sacral screw pelvic fixation in patients receiving either posterior spinal fusion or minimally invasive fusionless surgery, incorporating the most recent and relevant references in the field, and demonstrating that this fixation technique is effective in this specific population.

## 5. Conclusions

Despite the high rate of infectious complications (17%), the ilio-sacral screw is an effective tool to treat frontal and sagittal pelvic obliquity and spinal deformity in neuromuscular scoliosis patients. The mechanical complication rate was lower than that of other pelvic fixation procedures, as described in the literature. Intraoperative navigation should minimize the risk of nerve root injury and facilitate revision or primary fixation in patients with disturbed sacropelvic anatomy.

## Figures and Tables

**Table 1:** Main demographic data and radiologic outcomes of the series.

	PSF	MIFS	PSF and MIFS	*p*
Patients, *n*	62	111	173	
Mean age, *years*	15 (12 to 19)	12 (6 to 19)	13.7 (6 to 19)	
Etiology				
Muscular dystrophy, *n*	4 (6.5%)	10	14	0.0554
Cerebral palsy, *n*	42 (67.7%)	71	102	0.617
Flaccid Paraplegia, *n*	7 (11.3%)	4	11	0.057
Spinal muscular atrophy, *n*	6 (9.7%)	13	19	0.681
Rett syndrom, *n*	2 (3.2%)	5	7	1
Myelomeningocele	1 (1.6%)	8	9	0.159
Infections, *n*	13 (21%)	16 (14.4%)	29 (16.7%)	0.02 *
Local debridment and antibiotics, *n*	12	14	26	
Hardware removal, *n*	1	2	3	
Mechanical complications, *n*	5 (8.1%)	9 (8.1%)	14 (8%)	0.091
Early mobilization of S1 screw, *n*	5	1	6	
S1 root irritation, *n*	0	6	6	
Connector failure, *n*	0	1	1	
Ilio-sacral screw skin prominence, *n*	0	1	1	
Preoperative pelvic obliquity, (°), mean	23	23	23	
Last FU pelvic obliquity improvement (°), mean	−10.2 (44%)	−17.8 (77%)	−14	
Preoperative Cobb angle, (°), mean	50.5	77	63.8	
Last FU Cobb correction improvement, (°), mean	−31.1 (61%)	−47.8 (62%)	−39.5	
Loss of frontal or sagittal correction	None	None	None	
Sitting posture and comfort improvement	All cases	All cases	All cases	

PSF = posterior spinal fusion; MIFS = minimally invasive fusionless surgery; FU = follow-up; * Statistically significant.

## Data Availability

All data are available on demand from the corresponding author. The data are not publicly available because of privacy concerns.
